# Letter to Editor: Oral lesions in a patient with Covid-19

**DOI:** 10.4317/medoral.24044

**Published:** 2020-06-10

**Authors:** Ciro Dantas Soares, Rejane Andrade de Carvalho, Kalline Andrade de Carvalho, Maria Goretti Freire de Carvalho, Oslei Paes de Almeida

**Affiliations:** 1 DDS, Ph.D. Department of Oral Diagnosis, Piracicaba Dental School, University of Campinas, Piracicaba, SP, Brazil; 2 DDS, Ph.D. Department of Endodontics, Federal University of Rio Grande do Norte (UFRN), Natal, RN, Brasil.; 3 M.D. Brazilian Dermatology Society member. Department of Dermatology, Federal University of São Paulo (UNIFESP), São Paulo, SP, Brazil; 4 M.D., Ph.D. Laboratório de Citopatologia, Private Service, Natal, RN, Brazil

The coronavirus disease 2019 (COVID-19) is a global pandemic burden caused by the severe acute respiratory syndrome coronavirus 2 (SARS-CoV-2) infection with variable clinical outcome. The symptoms of this disease include headache, sore throat, fever, and dyspnoea. About 10% of the patients develop severe acute respiratory syndrome and 1-2 %, particularly elderly, die (1,2). Possible oral-related symptoms include hypogeusia, xerostomia and chemosensory alterations (3).

We report here the clinical and microscopical characteristics of oral reddish lesions and ulceration that occurred in a 42-year-old male patient positive for SARS-Cov-2 confirmed by polymerase chain reaction (PCR). The patient also had a history of diabetes and hypertension, and when admitted to the hospital presented fever (temperature of up to 39.3°C), cough and shortness of breath. On the skin it was observed some petechia-like and small vesicobullous lesions of unknown aetiology. A treatment with dexamethasone and dipyrone was established for 1 week.

**Figure 1:**
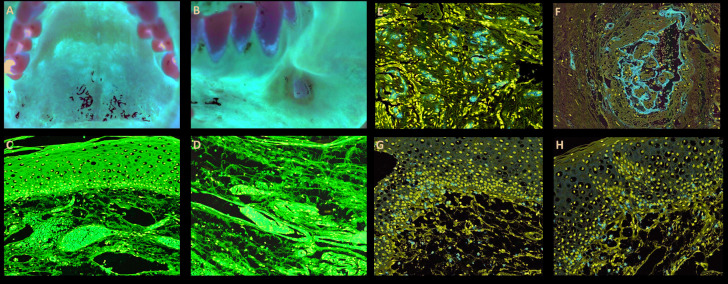
Clinical and microscopic features of reddish oral lesions in a patient with Covid-19. A: Diffuse reddish lesion in the hard palate. B: Ulcer with ischemic aspect in the buccal mucosa. C: Epithelium demonstrating vacuolization and haemorrhage in the superficial portion of the lamina propria, with hyperaemic vessels. D: Lymphocytic infiltration in the connective tissue and different-sized thrombi. E: CD34 positive expression in thrombi of small vessels. F: Larger thrombi with variable amount of fibrin and endothelial cells positive for CD34. G: CD3 positivity in most inflammatory cells in the connective tissue and basal layer of the epithelium. H: CD8 highlighting lymphocytes in the lamina propria and basal layer of the epithelium.

The patient also complained of a painful ulceration in the buccal mucosa that was biopsied. Oral examination showed besides the ulcerated lesion, multiple reddish macules of different sizes scattered along the hard palate, tongue, and lips (Fig. 1). After 3 weeks of follow up the lesions presented complete remission.

Microscopically the biopsied lesion, presented the epithelium with severe vacuolization and occasional exocytosis (Fig. 1). In the lamina propria, a diffuse chronic inflammatory infiltrate was associated with focal areas of necrosis and haemorrhage. Conspicuous superficial and deep small vessels were obliterated by evident thrombi (Fig. 1). Small thrombi seemed to be composed mainly by endothelial cells, while the larger ones were composed by fibrin and endothelial cells, and in either cases CD34 was positive for cells in the luminal component (Fig. 1). Adjacent minor salivary glands exhibited an intense lymphocytic infiltration, mostly positive for CD3 (Fig. 1) and CD8 (Fig. 1), and some of these cells were also found in the basal layer of the epithelium. Immunohistochemical reactions against HHV-1, HHV-2, CMV, treponema pallidum and EBV by in-situ hybridization were all negative. CD20, CD68, CD163, CD138 and CD4 demonstrated light focal positivity for the inflammatory cells. Considering the clinical and microscopical characteristics it was suggested that the patient presented lesions that could be associated with Covid-19 disease.

Diffuse thrombotic disease in the lung of patients with COVID-19 has been previously reported and seems to be common (4). To date, more than 5 million people were affected by COVID-19; however, scarce cases with possible oral manifestations have been reported (3,5). Therefore, oral involvement seems to be very rare. Herein, we report the clinical and microscopic aspects of oral lesions seen in a COVID-19 positive patient, which exhibited microscopically areas of haemorrhage and small thrombotic vessels. To the best of our knowledge, this is the first report of a patient positive for COVID-19 including the clinical and microscopical characteristics of oral lesions. These lesions must be better understood and characterized, considering the possible mechanisms involved. Based on clinical manifestations of this case and cases previously reported (5), the microscopic alterations may suggest a primary reaction to the SARS-CoV-2, as our patient did not have undergone intubation or any other traumatic event.

In short, we suggest that SARS-COV-2 can cause oral lesions and therefore all patients positive for the virus should have a full mouth examination to better understand the pathobiology of these oral alterations.
